# Asciminib, a novel allosteric inhibitor of BCR‐ABL1, shows synergistic effects when used in combination with imatinib with or without drug resistance

**DOI:** 10.1002/prp2.1214

**Published:** 2024-06-21

**Authors:** Naoki Okamoto, Kenta Yagi, Sayaka Imawaka, Mayu Takaoka, Fuka Aizawa, Takahiro Niimura, Mitsuhiro Goda, Koji Miyata, Kei Kawada, Yuki Izawa‐Ishizawa, Satoshi Sakaguchi, Keisuke Ishizawa

**Affiliations:** ^1^ Department of Clinical Pharmacology and Therapeutics Tokushima University Graduate School of Biomedical Sciences Tokushima Japan; ^2^ Department of Pharmacy Tokushima University Hospital Tokushima Japan; ^3^ Clinical Research Center for Developmental Therapeutics Tokushima University Hospital Tokushima Japan; ^4^ Department of Clinical Pharmacy Practice Pedagogy Tokushima University Graduate School of Biomedical Sciences Tokushima Japan; ^5^ Department of General Medicine Taoka Hospital Tokushima Japan; ^6^ Department of Respiratory Medicine and Rheumatology Tokushima University Graduate School of Biomedical Sciences Tokushima Japan

**Keywords:** asciminib, BCR‐ABL inhibitors, chronic myelogenous leukemia, drug resistance

## Abstract

In the treatment of chronic myeloid leukemia (CML), resistance to BCR‐ABL inhibitors makes it difficult to continue treatment and is directly related to life expectancy. Therefore, asciminib was introduced to the market as a useful drug for overcoming drug resistance. While combining molecular targeted drugs is useful to avoid drug resistance, the new BCR‐ABL inhibitor asciminib and conventional BCR‐ABL inhibitors should be used as monotherapy in principle. Therefore, we investigated the synergistic effect and mechanism of the combination of asciminib and imatinib. We generated imatinib‐resistant cells using the human CML cell line K562, examined the effects of imatinib and asciminib exposure on cell survival using the WST‐8 assay, and comprehensively analyzed genetic variation related to drug resistance using RNA‐seq and real‐time PCR. A synergistic effect was observed when imatinib and asciminib were combined with or without imatinib resistance. Three genes, GRRP1, ESPN, and NOXA1, were extracted as the sites of action of asciminib. Asciminib in combination with BCR‐ABL inhibitors may improve the therapeutic efficacy of conventional BCR‐ABL inhibitors and prevent the development of resistance. Its dosage may be effective even at minimal doses that do not cause side effects. Further verification of this mechanism of action is needed. Additionally, cross‐resistance between BCR‐ABL inhibitors and asciminib may occur, which needs to be clarified through further validation as soon as possible.

## Graphical abstract

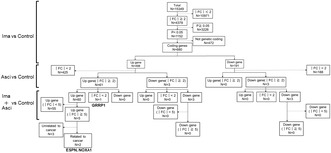

**Abbreviations**
CML: chronic myeloid leukemia
PI: propidium iodide
S.E.: standard error

## 1 INTRODUCTION

Chronic myelogenous leukemia (CML) is a type of myeloproliferative disease that affects approximately 1 in 100 000 individuals. The Philadelphia chromosome, formed by the translocation of chromosomes 9 and 22 in hematopoietic stem cells, causes this disease. It produces the BCR‐ABL protein that acts as a tyrosine kinase, leading to an overgrowth of white blood cells.^1^

CML prognosis considerably improved in therapeutic outcomes with the release of the BCR‐ABL inhibitor imatinib. Second‐generation BCR‐ABL inhibitors nilotinib and dasatinib were developed and led to an earlier complete molecular response and improved efficacy.^2^ Currently, third‐ and fourth‐generation agents are available, and the 5‐year survival rate for CML is >90%, offering long‐term survival prospects.^3^, ^4^

Withdrawal of BCR‐ABL inhibitors after patients have achieved complete remission results in relapse in approximately 60% of patients.^5^ Therefore, patients with CML require lifelong BCR‐ABL inhibitor treatment.^1^, ^6^ However, there are limited BCR‐ABL inhibitors, including imatinib, and when none show efficacy, patients may require transplantation.^7^ Transplantation has a lower life expectancy than BCR‐ABL inhibitors, along with numerous long‐term side effects. Therefore, we are developing strategies to prolong the use of existing BCR‐ABL inhibitors.

The primary cause of the reduced efficacy of BCR‐ABL inhibitors is the development of resistance. Factors contributing to resistance include point mutations in *BCR‐ABL* and abnormal expression of other genes.^8^ First, point mutations in *BCR‐ABL* alter the structure of the BCR‐ABL protein, preventing BCR‐ABL inhibitor binding to the BCR‐ABL protein.^9^ More than 90 different gene sites with point mutations have been identified, with the T315I mutation being the most severe and drug‐resistant.^10^ A point mutation replaces a single nucleotide, thereby changing the structure of the ATP‐binding pocket of BCR‐ABL1. This conformational change causes resistance to BCR‐ABL inhibitors because tyrosine kinase inhibitors are unable to bind to the ATP‐binding pocket.^11^

Ponatinib, a BCR‐ABL inhibitor that also shows efficacy against T315I, has been developed to address point mutation resistance, including T315I mutations; however, its efficacy is limited as it, like imatinib, targets the ATP‐binding site. Therefore, drug resistance due to point mutations remains unaddressed.^12^

Asciminib is a new and effective treatment for BCR‐ABL‐resistant CMLs. Asciminib has a different binding site for the BCR‐ABL protein compared to existing BCR‐ABL inhibitors.^13^ Therefore, it is predicted to maintain its efficacy in CML with specific point mutations such as T315I.^13^, ^14^ However, detailed validation is lacking.

The efficacy of BCR‐ABL inhibitors is also reduced by aberrant expression of genes other than the *Bcr‐Abl*.^15^ In particular, the aberrant expression of drug excretion transporters is believed to be a contributory factor in reducing intracellular drug concentration.^16^

Combining molecular targeted agents with similar sites of action can produce synergistic effects and delay the development of drug resistance seen with single agents.^17^, ^18^ Similarly, a combination of asciminib and imatinib, which have different sites of action, may produce synergistic effects. In fact, the benefit of asciminib in combination with Bcr‐Abl inhibitors has begun to be tested and may be useful for CML with point mutations.^19^ However, the detailed mechanism of resistance has not been determined and no effective therapy has been found for patients who do not have a genetic mutation yet are resistant to BCR‐ABL inhibitors.^15^

Thus, we examined the synergistic effect of the combination of asciminib and imatinib in BCR‐ABL inhibitor‐resistant CML and examined the mechanism of the synergistic effect.

## 2 METHODS

### 2.1 Reagents

imatinib was obtained from Tokyo Chemical Industry Co., Ltd., Japan. Asciminib was obtained from Cayman Chemical Company, USA. imatinib and ascribed were dissolved in dimethyl sulfoxide.

### 2.2 Cell culture

K562 cells (a CML cell line) were obtained from RIKEN BioResource Research Center, Japan. Cells were cultured in RPMI 1640 (Nacalai Tesque, Japan) supplemented with 10% fetal bovine serum (Biowest, USA) and 10 mg/mL 1% (v/v) penicillin–streptomycin (Wako, Japan). All cells were cultured at 37°C in a humidified atmosphere containing 5% (v/v) CO_2_. To develop imatinib‐resistant cell lines, imatinib concentrations were cultured in stepwise increments of 50–500 nmol/L over 3 months.

### 2.3 Cell survival assay

Cell survival assays were performed using a Cell Counting Kit‐8 (DOJINDO, Japan) according to the manufacturer's protocol. Cells (5000 cells/well) were seeded in 96‐well microplates and treated with or without the drug for 72 h at 37°C under humidified 5% (v/v) CO_2_. Thereafter, Cell Counting Kit‐8 was added at 10 μL/well and incubated for 3 h. Absorbance was measured at 450 nm (reference wavelength: 630 nm) using a microplate reader Model 680 (Bio‐Rad Laboratories, USA). Cell viability was calculated using the ratio of the absorbance of treated cells to that of the control (100%) with the following formula: viability = (absorbance of treated well − absorbance of blank well)/(absorbance of untreated well − absorbance of blank well) × 100.

The following equation was used to calculate the IC_50_ of each drug:

$${\mathrm{IC}}_{50}=10\frac{\left\{\log\left(A/B\right)\right\}\times \left(50-C\right)}{\left(D-C\right)+\log B}.$$

A: high concentration across 50%; B: low concentration across 50%; C: cell viability at B; and D: cell viability at A.

### 2.4 Gene expression analysis

The cells were seeded at 5.0 × 10^4^ cells/mL and divided into four groups (control, imatinib (125 nM), asciminib (3 nM), and imatinib (125 nM) + asciminib (3 nM) groups) and incubated in a 5% CO_2_ incubator at 37°C for 24 h. Thereafter, RNA was extracted using the RNeasy® Plus Mini Kit (QIAGEN, USA) according to the manufacturer's protocol and processed using the PrimeScript™ RT reagent Kit with gDNA Eraser (Perfect Real Time, Takara, Japan). A PCR Thermal Cycler Dice (Takara) was used for gDNA removal and reverse transcription. Thereafter, the StepOnePlus system (Applied Biosystems, Japan) was used to perform real‐time PCR on each reverse‐transcribed sample using the THUNDERBIRD SYBR qPCR Mix (Toyobo, Japan). Gene expression was quantified via relative expression analysis using the ΔΔCt method with hGAPDH as the internal standard.

### 2.5 Analysis of the cell cycle and apoptosis

K562 cells were seeded at a density of 5.0 × 10^4^ cells/mL and incubated with various agents for 72 h. For cell cycle analysis, cells were collected, washed with PBS, and resuspended in 70% ice‐cold ethanol overnight at −20°C. After the cells were centrifuged, the ethanol was discarded, and the cells were resuspended in 50 μL PBS containing 0.1 mg/mL RNase (F. Hoffmann‐La Roche, Ltd., Basel, Switzerland) for 30 min at 37°C. After washing with PBS, the cell pellet was incubated in 100 μL propidium iodide (PI) solution (BioLegend, USA) for 10 min and gently resuspended in the dark at 4°C. Apoptosis was measured using an Annexin V‐FITC Apoptosis Detection Kit (Nacalai Tesque) according to the manufacturer's protocol. Measurements were performed using a Cell Sorter SH800S (Sony, Japan). Kaluza software (Beckman Coulter, USA) was used for the analysis.

### 2.6 RNA sequencing

RNA sequencing analysis was performed using RNA samples generated during gene expression analysis. BCR‐ABL point mutation analysis was performed by Eurofins, Japan, and RNA‐seq analysis was performed by Macrogen, Japan.

### 2.7 Statistical analysis

Data were expressed as the mean ± S.E. (standard error) for each value. The sample size “*n*” indicates the number of independent experiments performed on independent plates. Statistical analysis was performed using one‐way ANOVA or Tukey's multiple comparisons among the three groups using IBM SPSS Statistics 29 software. Statistical differences between the two groups were evaluated using Student’s *t*‐test.

### 2.8 Nomenclature of targets and ligands

Key protein targets and ligands in this article are hyperlinked to corresponding entries in http://www.guidetopharmacology.org, the common portal for data from the IUPHAR/BPS Guide to PHARMACOLOGY,^20^ and are permanently archived in the Concise Guide to PHARMACOLOGY 2019/20.^21^

## 3 RESULTS

### 3.1 Asciminib shows synergistic effects when combined with imatinib

**FIGURE 1:**
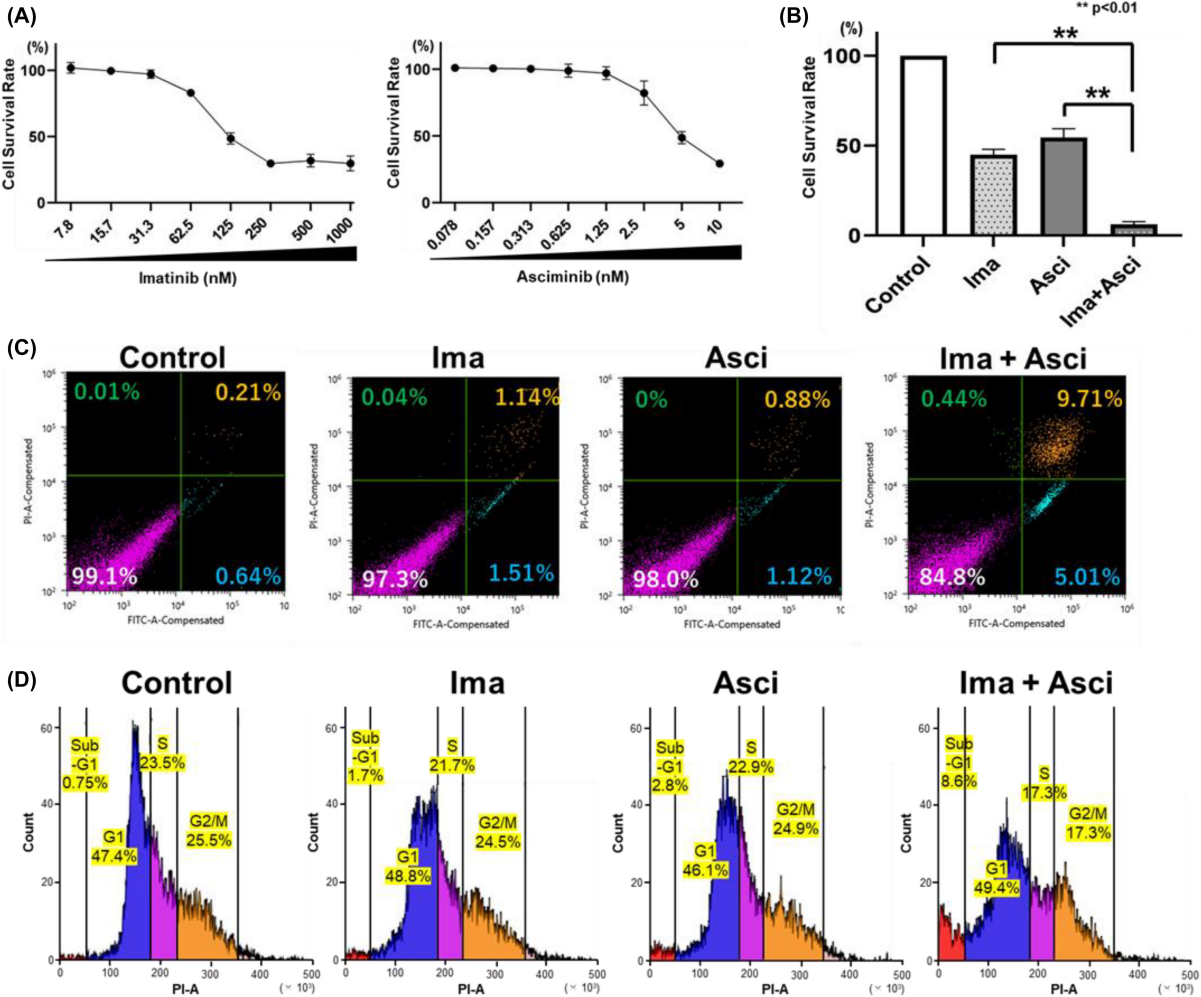
The effect of imatinib or asciminib on cell survival and promotion of angiogenesis. K562 cells, a BCR‐ABL‐positive CML cell line, were used to investigate drug sensitivity. Cells (1 × 10^5^ cells/well) were plated in 96‐well plates with (A) imatinib (7.8–1000 nM), asciminib (0.078–10 nM), (B) imatinib (125 nM), and asciminib (3 nM). After 72 h of incubation, cell viability was determined using the WST‐8 assay. Error bars represent the mean ± SE (*n* = 3). (C) asciminib enhanced imatinib‐induced apoptosis in BCR‐ABL‐positive CML cells. Cells were cultured with imatinib (125 nM) and asciminib (3 nM) for 24 h and subsequently examined for apoptosis using annexin V and PI staining. (D) asciminib enhanced imatinib‐induced cell cycle arrest in BCR‐ABL‐positive CML cells. Cells were cultured with imatinib (125 nM) and asciminib (3 nM) for 24 h and subsequently examined for cell cycle using PI staining.

Changes in the proliferation of K562 cells exposed to different doses of imatinib or asciminib were evaluated using the WST‐8 assay. K562 cell proliferation was inhibited by imatinib and asciminib in a dose‐dependent manner, with IC_50_ values of 121 nM and 4.9 nM, respectively (Figure 1A). Simultaneous exposure to imatinib and asciminib resulted in greater inhibition of cell proliferation than a single exposure (Figure 1B). Furthermore, annexin V and PI were used to determine the status of cell death. Apoptosis was detected when imatinib and asciminib were used in combination and when they were used individually (Figure 1C). Similarly, in the cell cycle, imatinib and asciminib exposure increased the percentage of cells in the sub‐G1 phase, and the combination of the two drugs further increased this percentage in the sub‐G1 phase (Figure 1D).

### 3.2 Asciminib shows synergistic effects when combined with imatinib, even among imatinib‐resistant lines

**FIGURE 2:**
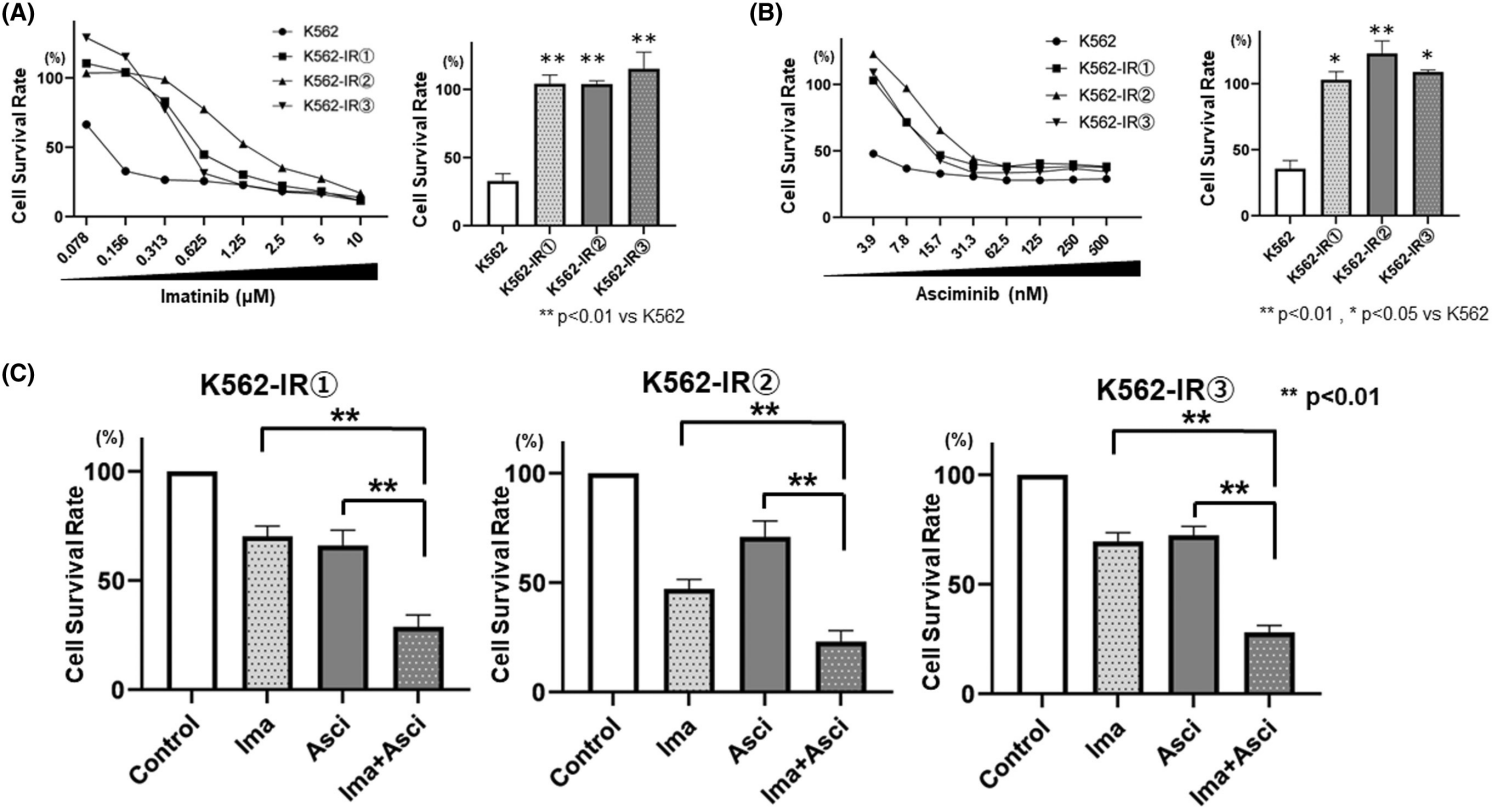
The effect of imatinib or asciminib on cell survival and promotion of angiogenesis in imatinib‐resistant cell lines. K562 and three K562‐IR cell types (density: 1 × 10^5^ cells/well) were plated in 96‐well plates with (A) imatinib or (B) asciminib or (C) imatinib and asciminib, at their respective IC_50_ values. Treatment concentrations were imatinib 600 nM and asciminib 15 nM for (1), imatinib 1400 nM and asciminib 25 nM for (2), and imatinib 500 nM and asciminib 15 nM for (3). After 72 h of incubation, cell viability was determined using the WST‐8 assay. Error bars represent the mean ± SE (*n* = 3).

The established imatinib‐resistant K562 cells (K562‐IR1, K562‐IR2, and K562‐IR3) were used for validation. Cells were exposed to different doses of imatinib or asciminib for 72 h, and the inhibition of cell proliferation was evaluated using the WST‐8 assay. Cell proliferation was inhibited in a concentration‐dependent manner by both imatinib and asciminib; the IC_50_ values for imatinib in imatinib‐resistant K562‐IR1, K562‐IR2, and K562‐IR3 cells were 569, 1386, and 472 nM, respectively (Figure 2A). The IC_50_ for asciminib in all imatinib‐resistant K562 cells (K562‐IR1, K562‐IR2, and K562‐IR3) was higher than that of the non‐resistant cells, with values of 14.3 nM, 26.1 nM, and 13.2 nM, respectively (Figure 2B). Moreover, concomitant exposure to imatinib and asciminib inhibited cell proliferation to a greater extent than single‐agent exposure (Figure 2C).

### 3.3 Confirmation of resistance mechanism of resistant cell lines

**FIGURE 3:**
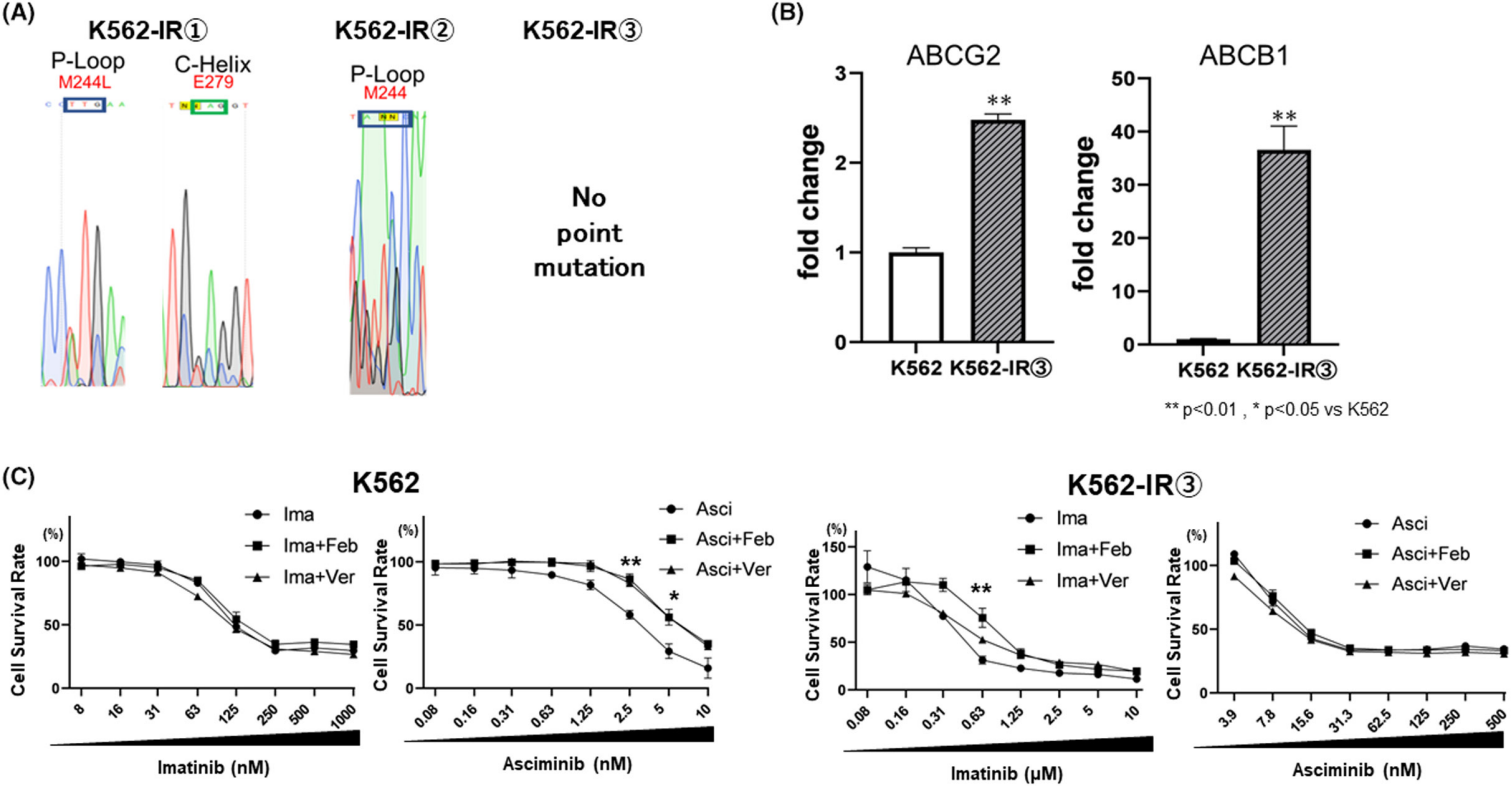
Contribution of point mutations and drug excretion transporters in the imatinib‐resistant K562 cell line. (A) RNA‐seq was performed on K562 cells for the presence of point mutations in the binding sites of Bcr‐Abl inhibitors. Point mutations were found in K562‐IR1 and K562‐IR2; however, no point mutations were found in K562‐IR3. (B) Expression of drug excretion transporter in K562‐IR3 cells (primers are noted in Supply). (C) Validation was conducted in both K562 and K562‐IR3 cells to assess changes in cell viability when exposed to imatinib and asciminib in the presence of drugs inhibiting ABCG2 or ABCB1 were measured. Cell viability was measured after 72 h of drug exposure and 72 h of culture using the WST‐8 assay. Error bars represent the mean ± SE (*n* = 3).

The status of point mutations in the established imatinib‐resistant K562 cells was confirmed by RNA sequencing analysis. Two point mutations were detected in K562‐IR1 and one in K562‐IR2; however, none were detected in K562‐IR3 (Figure 3A). Therefore, we focused on K562IR‐3 to identify resistance mechanisms other than point mutations. Using real‐time PCR, we confirmed that the expression of the drug excretion transporters ABCG2 and ABCB1 was upregulated in resistant cells compared to that in non‐resistant cells (Figure 3B). Next, we used febuxostat and verapamil, inhibitors of ABCG2 and ABCB1, respectively, to confirm the effects of ABCG2 and ABCB1 inhibition on the survival of K562‐IR3 cells.

Exposure to either febuxostat or verapamil in the presence of imatinib or asciminib did not enhance cell growth inhibition compared to that in the imatinib‐alone group (Figure 3C).

### 3.4 Comprehensive analysis via RNA‐seq

**FIGURE 4:**
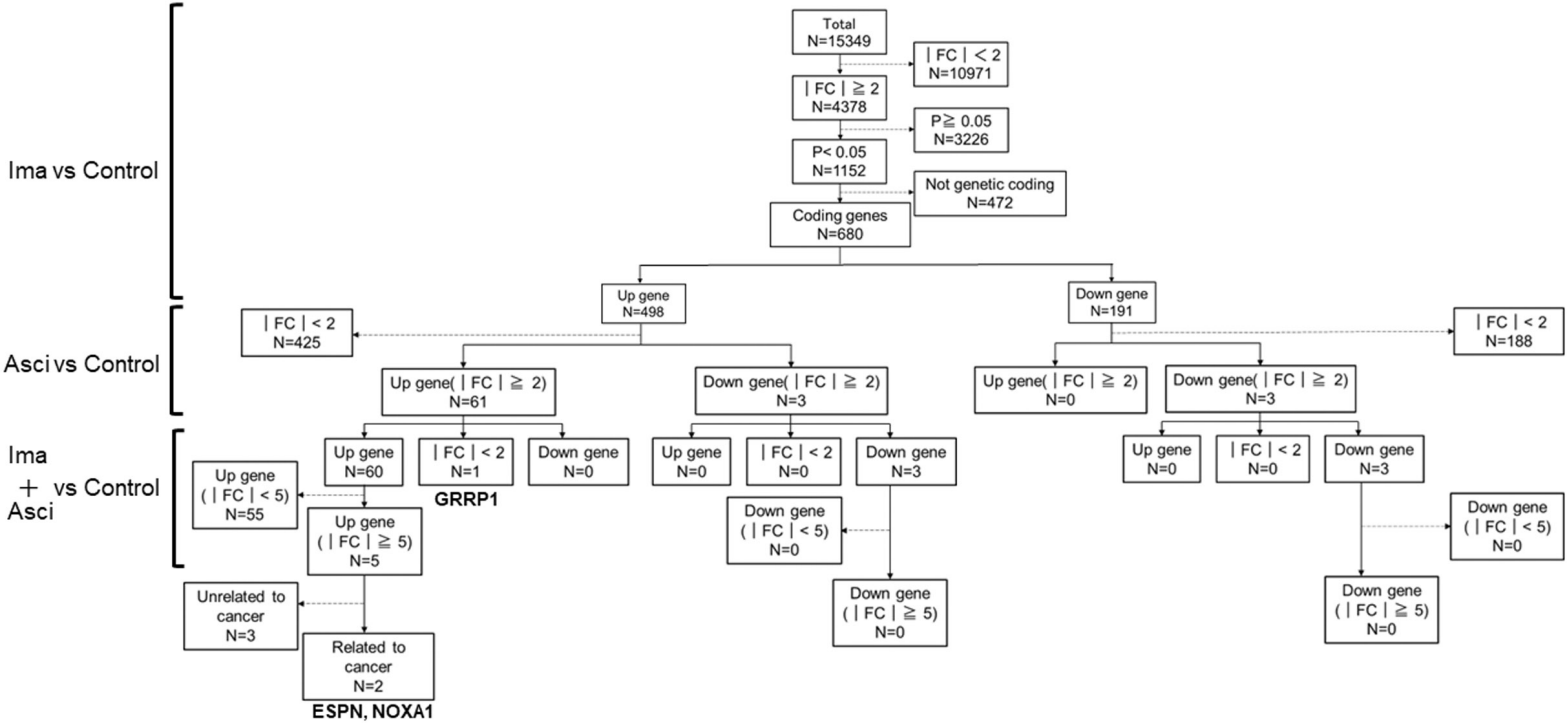
Comprehensive analysis of genes associated with imatinib and/or asciminib. RNA‐seq analysis was performed on K562 cells treated with imatinib (125 nM) and asciminib (3 nM) for 24 h and extracted RNA samples. A total of 15 349 genes were included in the analysis. Of these, 1152 genes had a significant twofold or greater change in gene expression due to imatinib exposure. *n* = the number of genes included. Among them, 680 gene‐encoding proteins were validated. Then, genes whose expression was upregulated more than twofold in both imatinib and asciminib groups compared to the untreated group were included in the analysis, and 61 genes were applicable. Furthermore, with regard to the variation when imatinib was combined with asciminib, 2 genes whose expression was upregulated were reported to be associated with cancer, and 3 genes whose expression was not yet reported. Thresholds are indicated by FC and the amount of variation compared to the drug untreated group. For the amount of variation, *p* < .05 is indicated as a significant variation.

RNA‐seq analysis was performed on K562 cells treated with imatinib, asciminib, and their two‐drug combination. Genes encoding these proteins have been explored for their association with cancer. We analyzed 15 349 genes. Three genes were selected: GRRP1, upregulated by both imatinib and asciminib, and ESPN and NOXA1, upregulated by both imatinib and asciminib (Figure 4).

### 3.5 Comprehensive analysis via RNA‐seq

**FIGURE 5:**
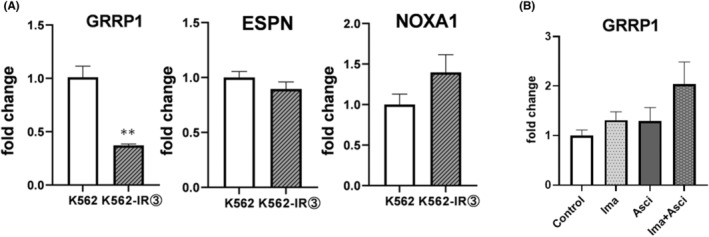
Expression levels for a group of genes that may be related to drug resistance. (A) Gene expression of GRRP1 in K562 and K562‐IR3 cells was tested for differences in gene expression of GRRP1 in cells that developed drug resistance. Error bars represent mean ± SE (*n* = 3). (B) Gene expression of GRRP1 was analyzed using RNA samples extracted after culturing cells in imatinib (125 nM) and asciminib (3 nM) for 24 h in K562. Error bars represent mean ± SE (*n* = 3).

For the three genes extracted by RNA‐seq, the variation in expression levels in drug‐resistant strains was verified by real‐time PCR using K562 and K562‐IR3. For GRRP1, expression was found to be decreased in resistant cells compared to non‐resistant cells. On the other hand, no significant variation in expression was observed for ESPN and NOXA1 (Figure 5A). Therefore, we exposed K562 to imatinib and asciminib and confirmed the variation of GRRP1 gene expression; the expression level tended to increase after exposure to imatinib and asciminib, and further increase was observed when both drugs were combined (Figure 5B).

## 4 DISCUSSION

BCR‐ABL inhibitors have significantly altered CML treatment.^2^, ^3^, ^4^ However, owing to drug resistance to BCR‐ABL inhibitors and other factors, some patients exhaust these options and require hematopoietic stem cell transplantation.^4^ Therefore, it is important to elucidate the mechanism of resistance to BCR‐ABL inhibitors.

In this study, although both imatinib and asciminib inhibited BCR‐ABL, no competitive antagonism was observed when both drugs were used together. Instead, synergistic effects were observed. The combination increased apoptotic cells in annexin V and PI analysis. In addition, cell cycle analysis using flow cytometry showed an increase in the percentage of cells in the sub‐G1 phase, indicating an increase in apoptotic cells.^22^, ^23^ These results supported the possibility that the combination of imatinib and asciminib does not induce substantial toxicity. The reason for the synergistic effect may be because the mechanism of BCR‐ABL inhibition by asciminib is different from that of existing BCR‐ABL inhibitors.^13^, ^14^ Additionally, the combination of two molecular targeted agents with a close point of action decreases the risk of developing resistance to these agents.^17^, ^18^ Although the current approval for asciminib and imatinib is limited to single‐agent use, their combination may reduce the risk of drug resistance compared with the single‐agent use of each agent. The optimal dose of asciminib when combined with the two drugs needs further validation. The risk of side effects may be low if the dosage of asciminib is small when compared with the currently indicated dosage.

Resistance to BCR‐ABL inhibitors is another major challenge in CML treatment. Factors contributing to resistance to BCR‐ABL inhibitors include point mutations in *BCR‐Abl* and abnormal expression of all other genes.^8^ asciminib, with a distinct mechanism of action from conventional BCR‐ABL inhibitors, is not expected to induce resistance, even in the case of point mutations.^14^ Therefore, asciminib has been used in clinical practice for patients with CML who show resistance to BCR‐ABL inhibitors.^24^ Thus, we established multiple imatinib‐resistant K562 cell lines and tested their sensitivity to asciminib.

First, we examined sensitivity to asciminib alone, observing decreased sensitivity in all imatinib‐resistant cells to asciminib compared to non‐resistant strains. Although the combination with asciminib did not restore sensitivity to imatinib, the synergistic effect of the combination was confirmed. These results suggested that greater efficacy may have been achieved if asciminib alone or in combination with a BCR‐ABL inhibitor had been used in the same patient at the initial onset of CML, before BCR‐ABL inhibitor resistance development. Based on these results, further validation is essential, including clinical practice verification of cross‐resistance, comparing asciminib resistance in samples taken at the time of initial CML onset with that in samples taken at the time of resistance to BCR‐ABL inhibitors.

Point mutations may not be involved in the development of drug resistance. Therefore, we further investigated K562‐IR3, an established resistant strain lacking a point mutation. Activation of the drug excretion transporters ABCB1 and ABCG2 is a typical cause of drug resistance to BCR‐ABL inhibitors.^25^, ^26^ We observed that the expression of these genes was upregulated in K562‐IR3. However, exposing K562‐IR3 cells to the inhibitors of ABCB1, verapamil,^27^ and the inhibitor of ABCG2, febuxostat,^28^ did not restore sensitivity to imatinib or asciminib. Therefore, it is unlikely that ABCB1 or ABCG2 contributes to asciminib or imatinib resistance in K562‐IR3 cells. In cells that develop resistance to a molecularly targeted drug, the use of the drug may reverse target activation.^29^ In this study, using ABCG2 or ABCB1 inhibitors increased cell viability compared to the untreated group. This may be due to the activation of the signaling pathway resulting from increased intracellular concentrations of imatinib and asciminib. Since these observations were verified in basic research, further investigation using clinical samples is planned to determine whether they also occur in clinical patients.

Next, RNA‐seq analysis was performed to investigate the mechanism of the synergistic effect of imatinib and asciminib, considering asciminib is a new drug and the majority of its secondary effects are unknown. This may contribute to understanding the mechanism by which CML cells acquire drug resistance to imatinib. Therefore, we performed a comprehensive search for protein‐coding genes using RNA‐seq. Three genes, GRRP1, ESPN, and NOXA1, were identified as active sites. These genes are associated with cancer growth.^30^, ^31^, ^32^, ^33^ However, there are no related reports on asciminib, imatinib, or CML, which may be novel sites of action that show synergistic effects.

Therefore, we examined the expression levels of GRRP1 in drug‐resistant cells and found that GRRP1 expression was significantly downregulated in resistant cells compared to non‐resistant cells, while no significant changes in expression were observed for ESPN and NOXA1. Thus, we exposed imatinib and asciminib to K562 and confirmed the changes in GRRP1 gene expression. The expression levels tended to increase after exposure to imatinib and asciminib, and a further increase was observed when both drugs were used in combination. These results suggest that GRRP1 may be involved in the synergistic effects of imatinib and asciminib and in drug resistance.

In this study, the use of asciminib in combination with Bcr‐Abl inhibitors improved the therapeutic efficacy of conventional BCR‐ABL inhibitors and prevented the development of resistance. It may be effective even at minute doses without causing any side effects. In addition, cross‐resistance may occur between BCR‐ABL inhibitors and asciminib, and it may be important to use asciminib from the initial stage of the disease before the development of resistance. However, whether cross‐resistance to BCR‐ABL inhibitors occurs when resistance develops to asciminib remains unclear, necessitating further validation. We found GRRP1 as a new point of action involved in these drug resistance and synergistic effects. GRRP1 is involved in the induction of cell differentiation,^31^ and decreased GRRP1 expression may have increased the number of undifferentiated cells, resulting in decreased drug sensitivity. Although further validation of the detailed mechanism of action of GRRP1 is needed, GRRP1 may be a valuable point of action in the treatment of CML.

## AUTHOR CONTRIBUTIONS

KY, FA, and KI wrote the manuscript; KY, SI, and SS designed the research; KY, SI, MT, FA, TN, KM, and YI performed the research; and KY, SI, MT, MG, KK, NO, and KI analyzed the data.

## FUNDING INFORMATION

This study was supported by a Grant‐in‐Aid (KAKENHI) from the Japan Society for the Promotion of Science (JSPS) (grant numbers: 20 K16044 and 22 K15340). The funding source had no role in the study design; collection, analysis, and interpretation of data; writing of the report; or decision to submit the article for publication.

## DECLARATIONS OF INTEREST

None.

## Supporting information


**Table S1:** xxx

## Data Availability

Data pertaining to this article will be available by the corresponding author upon reasonable request.
